# Rewarding imperfect motor performance reduces adaptive changes

**DOI:** 10.1007/s00221-015-4540-1

**Published:** 2016-01-12

**Authors:** K. van der Kooij, K. E. Overvliet

**Affiliations:** Department of Behavioural and Human Movement Sciences - Research Institute MOVE, Vrije Universiteit, Van der Boechorststraat 9, 1081 BT Amsterdam, The Netherlands; Department of Biological Psychology and Neuropsychology, University of Hamburg, Von-Melle-Park 11, 20146 Hamburg, Germany

**Keywords:** Visuomotor adaptation, Motor adaptation, Error-based learning, Reinforcement learning, Reward

## Abstract

Could a pat on the back affect motor adaptation? Recent studies indeed suggest that rewards can boost motor adaptation. However, the rewards used were typically reward gradients that carried quite detailed information about performance. We investigated whether simple binary rewards affected how participants learned to correct for a visual rotation of performance feedback in a 3D pointing task. To do so, we asked participants to align their unseen hand with virtual target cubes in alternating blocks with and without spatial performance feedback. Forty participants were assigned to one of two groups: a ‘spatial only’ group, in which the feedback consisted of showing the (perturbed) endpoint of the hand, or to a ‘spatial & reward’ group, in which a reward could be received in addition to the spatial feedback. In addition, six participants were tested in a ‘reward only’ group. Binary reward was given when the participants’ hand landed in a virtual ‘hit area’ that was adapted to individual performance to reward about half the trials. The results show a typical pattern of adaptation in both the ‘spatial only’ and the ‘spatial & reward’ groups, whereas the ‘reward only’ group was unable to adapt. The rewards did not affect the overall pattern of adaptation in the ‘spatial & reward’ group. However, on a trial-by-trial basis, the rewards reduced adaptive changes to spatial errors.

**Electronic supplementary material:**

The online version of this article (doi:10.1007/s00221-015-4540-1) contains supplementary material, which is available to authorized users.

## Introduction

Rewarding imperfect performance is commonplace in training processes. Children are praised for their first unstable steps, and sports competitions are held against competitors of equal strength such that imperfect movements may still be rewarded with the experience of winning. Rewarding imperfect performance makes sense because being rewarded fulfills the psychological need of competence, even when the reward is fictitious (Deci and Ryan 2000; Przybylski et al. 2010). Reward may therefore seduce the learner to engage in more practice. Hence, reward systems such as scored points or collected badges are added to game-like protocols for motor learning in rehabilitation (Granic et al. 2013; Hamari et al. 2014; Mayer et al. 2013). Although receiving rewards for performances that are obviously not flawless is commonplace, very little is known on whether and how rewards affect the manner in which people learn from their errors (Galea et al. 2015).

Here, we focus on motor adaptation, which is often studied in a ‘visuomotor rotation paradigm’ in which participants make reaching movements toward (virtual) visual targets while learning to correct for a rotation of visual feedback about the hands direction (e.g., Burge et al. 2008; Cheng and Sabes 2007; Galea et al. 2015, 2010; Hinder et al. 2010). Experiments using this paradigm have uncovered that rewards and spatial errors (deviations between the movement outcome and target) drive different learning systems that have been associated with different adaptation outcomes. Rewards provide input to a system that reinforces successful movements and learns slowly but has good retention (Huang et al. 2011; Izawa and Shadmehr 2011; Therrrien et al. 2015). Spatial errors on the other hand provide input to a learning mechanism that learns the mapping between visual targets and motor output by learning from a fraction of each spatial error. An implicit process is associated with fast learning but relatively poor retention (e.g., Baddeley et al. 2003; Burge et al. 2008; Hinder et al. 2010; Krakauer 2009; van Beers 2009), whereas an explicit process, finally, determines aiming strategies (Benson et al. 2011; Mazzoni and Krakauer 2006; Redding and Wallace 2002; Taylor et al. 2014) and has been associated with fast learning (Benson et al. 2011) and savings: Learning rates are faster during re-adaptation (Haith et al. 2015).

The reward- and error-based systems were initially considered to be independent (Huang et al. 2011; Izawa and Shadmehr 2011; Shmuelof et al. 2012), but recent results indicate that the combination of reward and (spatial) error feedback enhances motor adaptation, which raises the question whether these are additive effects or whether rewards modulate error-based learning. One study, using a visuomotor rotation task, found that the combination of financial reward and (spatial) error feedback enhances the retention of adaptation during an episode without error feedback (Galea et al. 2015). Another study also showed retention benefits but only after a night of sleep (Abe et al. 2011). Moreover, reward seems to induce faster learning (Abe et al. 2011; Dayan et al. 2014; Nikooyan and Ahmed 2015). However, other studies show positive effects of rewards only after taking away visual feedback (Shmuelof et al. 2012), or after making the participants aware of the purpose of rewards (Manley et al. 2014). For this mix of results, it remains unclear how the combination of rewards and spatial errors affects adaptation. There may be an additive effect of reward-based and error-based learning mechanisms (Shmuelof et al. 2012), but there may also be a modulatory effect of the rewards in which the rewards affect how participants learn from their spatial errors, for instance due to modulation of error sensitivity (Nikooyan and Ahmed 2015) or dopaminergic enhancement of memory signals (Abe et al. 2011; Galea et al. 2015).

In the present study, we examine the effect of rewards on visuomotor adaptation in a 3D version of the visuomotor rotation task. Learning and retention of the adaptation are assessed in alternating phases with and without performance feedback (spatial errors or a combination of spatial errors and reward). We focus on the modulatory effects of rewards on learning from spatial errors. To do so, we designed an adaptive reward scheme in such a way that about half of the trials is rewarded, regardless of the participants’ improvement at the task. This lead us to reward trials that involve significant spatial errors, allowing us to compare the learning of spatial errors between trials that have been rewarded and trials that have not been rewarded. Moreover, the rewards were binary and provided no information about the spatial distance from the targets. Together with their abundance, this makes it unlikely that the rewards provide a source of information that can be used independent of the spatial errors in reward-based reinforcement learning. Two main groups of participants were compared: a group that receives spatial feedback only and a ‘spatial & reward’ group, in which the adaptive reward scheme is added to the spatial feedback. To test whether rewards modulate adaptation, groups were compared on the asymptotic adaptation (in the learning and retention phases), the early adaptation and error sensitivity of their spatial errors.

## Methods

### Participants

Forty-six participants [23 males and 23 females, all right-handed, mean age 23.78 years (SD 3.91 years)] participated in the study. Participants had normal binocular vision as tested with the Randot *stereo fly* test (median stereo acuity of 40 s of arc). Twenty of the participants were randomly assigned to a ‘spatial only’ group and another 20 to a ‘spatial & reward’ group. After measuring the main experiment, 6 additional participants were recruited and assigned to a ‘reward only’ group. Participants either took part voluntarily (students and colleagues in the department) or got paid 8 euros (everyone else). Payment was independent of performance in the study and was provided by bank transfer in the weeks following the experiment. In the ‘spatial only’ group, 11 out of 20 participants participated voluntarily and in the ‘spatial & reward’ group 12 out of 20 participants participated voluntarily. The study was conducted in accordance with the Declaration of Helsinki and was part of an ongoing research program for which consent procedures were approved by the ethics committee of the Faculty of Human Movement Sciences of the VU University. All participants gave written informed consent by signing an informed consent document. All data were encoded and analyzed anonymously, and all participants were naïve to the purpose of the experiment.

### Setup

The setup was similar to the one used in earlier studies (van der Kooij et al. 2013, 2015) and is described below.

**Fig. 1:**
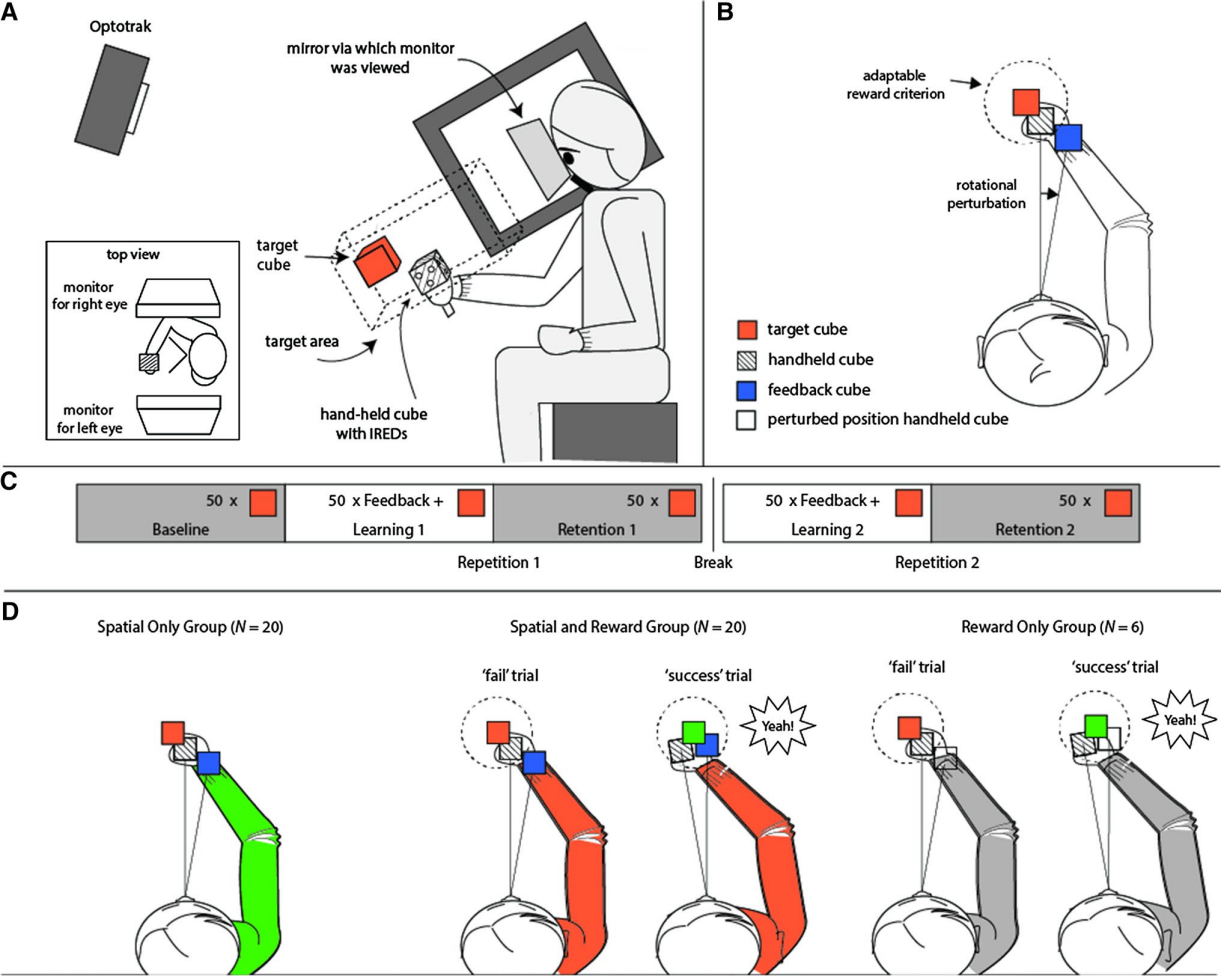
**a** The experimental setup (*side view*) with an *inset* showing the *top view* (see ‘Methods’ section for a detailed description). **b** Illustration of the rotational perturbation of the spatial feedback (*blue cube*) and the adaptable reward criterion (*dashed circle*). If the center of the (perturbed) feedback cube fell within the reward criterion, a trial was classified as a ‘success’ trial. Otherwise, it was classified as a ‘fail’ trial. Note that the reward criterion was spherical and that the illustration provides a *top view*. **c** Alternating phases of 50 trials without any feedback and in which only the targets were visible were alternated with blocks of 50 trials in which feedback was provided on the movement end position. **d** Illustration of the endpoint feedback provided in the three reward groups: left spatial only, middle spatial & reward, right reward only (color figure online)

Participants were seated in a light proof room, where they viewed two separate CRT displays (48 × 31 cm; viewing distance about 40 cm; resolution 1096 × 686 pixels, 160 Hz) with each eye via mirrors (Fig. 1a). Infrared emitting diodes (IREDs) were mounted on a cube with 5-cm edges with a handle that participants held in their right hand and that allowed us to track the movements of the participants’ hand at 100 Hz with an Optotrak 3020^®^ motion analysis system (NDI, Waterloo, ON, Canada). To be able to render an adequate image of the scene without having to restrain the participant’s head, IREDs were mounted on a bite board that participants held in their mouth and that was not connected to the setup. For each participant, we determined the eyes’ locations relative to the bite board in a calibration session (Sousa et al. 2010). This allowed us to render an appropriate new image of the 3D scene for each eye with a latency of ~25 ms between participants’ movements and the corresponding update of the display. As a result, this setup renders a realistic representation of the 3D space in front of the participant, without them being able to see their handholding the cube as they move from target to target.

### Task

Participants were positioned in the virtual reality setup and asked to align the position of the handheld cube with the position of red target cubes. Participants were explained the course of the experiment and told that they would see a representation of the handheld cube in blue at the end of each trial in the feedback blocks, but that in the other blocks they would not receive feedback at all. Target’s appeared one by one, within a (invisible) 10 by 10 by 30 cm target area elongated along the participants’ line of sight (Fig. 1a).

A trial started with the appearance of a new target. The first trial was presented at the center of the target area, and for the location of the subsequent targets, the target moved 20 cm in a random direction but a different angle was chosen if the new point was outside the target area. As soon as the new target was presented, the participant could initiate a movement toward the target. When the movement velocity dropped below 2 cm per second for 300 ms, the position of the handheld cube at that movement was registered as the movement endpoint. Depending on whether the experimental phase involved feedback, a new target appeared or feedback was provided. When feedback was provided, it was based on a perturbed end position that was rotated 10° in azimuth around the cyclopean eye (Fig. 1b). Hence, the movement endpoint had to be 10° azimuth counterclockwise of the target for the participant to receive feedback that the handheld cube and target were aligned.

Alternating phases without and with performance feedback formed three different experimental phases (Fig. 1c): In an initial ‘baseline’ phase without feedback, baseline performance was measured. In a subsequent ‘learning’ phase in which feedback was available, participants could adapt to the perturbed feedback. In a following ‘retention’ phase without feedback, we measured how much of the adaptation was retained in the absence of feedback. These learning and retention phases were repeated once, to measure savings of motor adaptation (faster re-adaptation to the perturbation).

The three experimental phases were performed by three groups of participants (Fig. 1d) that received either only spatial feedback (*N* = 20), a combination of spatial feedback and reward (*N* = 20), or reward only (*N* = 6). Spatial feedback was provided by means of a (static) blue cube that appeared for 500 ms at the rotated movement endpoint. Reward feedback was presented as a target color change, an accompanying ‘yeah’ sound and ten scored points that were added to the participants’ cumulative score that was displayed in the middle of the visual field. Before starting the experiment, participants in the groups that received reward were told that they were competing with the other participants. To ensure that a sufficient amount of trials was rewarded and to keep the proportion of rewarded trials constant between participants, the rewards were based on whether participants hit a spherical adaptive ‘hit area’ around the target. The diameter of this reward criterion depended on the participant’s performance. In the first five trials, the radius was defined as 300 mm, but from the 6th feedback trial onwards it was defined as the average absolute error (*U*) of the last five trials, if lower than the previous diameter. To ensure that the hit probability was relatively constant, the radius (*r*) of the hit area was increased with 10 mm when the participant had not been reinforced for 10 trials.

$$ \begin{aligned}& r_{(n = 1:5)} = 300 \hfill \\ &  r_{(n > 5)} = \bar{U}_{(n - 5:n - 1)} \hfill \\ & {\text{`Fail'}}\,{\text{trial}}{:}\,U_{n} > r_{n} \hfill \\ &  {\text{`Success'}}\,{\text{trial}}{:}\,U_{n} \le r_{n} \hfill \\ \end{aligned} $$

### Procedure

After being informed about the experimental procedures and signing an informed consent document, we calibrated the participants’ bite board. Next, the participant started with the baseline phase followed by a learning and retention phase. After that, participants took a short break to prevent arm fatigue. In this break, we informed participants in the spatial & reward condition about their score and encouraged them to improve their score of the first block. After the break, participants performed another learning and retention phase. In total, the experiment took about 20 min. The complete session including the instructions, calibration and breaks took around 45 min.

### Data analysis

As we imposed a perturbation in the azimuthal direction, we analyzed azimuthal errors (*θ*). Analyses of absolute errors (*U*) can be found in the supplementary materials. Azimuthal errors were defined as the difference between azimuthal direction of the target and azimuthal direction of the handheld cube at the movement endpoint. Outliers were calculated per trial and per feedback condition. Trials for which the azimuthal error differed from the mean with more than 2.5 times the standard deviation of the mean were considered outliers and were discarded. This resulted in the exclusion of 2.7 % of the trials. The amount and rate of adaptation are generally assessed by fitting a state space or exponential model to the data (e.g., Burge et al. 2008; Smith et al. 2006). However, the large response variability in the 3D pointing task results in unreliable model fits for individual participants. We therefore opted for model-free parameters.

The amount of adaptation was estimated from the adaptation asymptote, or the steady state pointing error after adaptation, which was defined as the mean azimuthal error over the last 25 trials of a learning or retention phase. The rate of adaptation was estimated from the early adaptation, which was defined as the mean azimuthal error on trial 2–6 of a learning phase as in (Huberdeau et al. 2015). To circumvent the possible effect of differences in baseline biases (van der Kooij et al. 2013), both parameters were calculated relative to the mean azimuthal error in trials 11–40 of the baseline phase.

Besides analyzing the overall adaptation, we also performed a more fine-grained analysis in which we compared the change in pointing angle (Δ*θ*)^1^ on success and fail trials. For this analysis, we calculated the change in pointing angle *θ* from trial *n* to trial *n* + 1:

$$ \Delta \theta^{(n)} = \theta^{(n + 1)} - \theta^{(n)} $$

To analyze how Δ*θ* depended on the combination of spatial error and reward, we binned Δ*θ* into the Δ*θ* for spatial errors that were smaller than the overall mean spatial error and Δ*θ* for spatial errors that were larger than the overall mean spatial error in the learning phases. To control for the fact that the rewarded errors were generally smaller and that smaller errors have been associated with greater error sensitivity (Criscimagna-Hemminger et al. 2010; Marko et al. 2012), we used the spatial only group as a control condition in the comparison of Δ*θ* for rewarded and non-rewarded errors. We therefore calculated the adaptive reward criterion *r* in the spatial only group as well as in the groups that received reward. This way we could compare Δ*θ* for success and fail trials with different spatial errors between the spatial & reward group in which the success trials were rewarded and the spatial only group in which there were no rewards.

Statistical analysis was performed using IBM SPSS version 22. Data from the main groups (‘spatial only’ vs. ‘spatial & reward’) and data from the control group (‘reward only’) were analyzed separately. The parameters were tested for normality using Shapiro–Wilkinson tests with a *p* value at 0.05. As the parameters passed this test, we used analysis of variance to test our hypotheses.

The effect of reward group on the adaptation asymptotes was analyzed in a mixed-model ANOVA with reward (spatial only, spatial & reward) as between-subjects factor and adaptation phase (learning, retention) and repetition (first vs. second) as within-subjects factors. For this analysis, we expected that participants adapt their spatial errors to the feedback, which would result in a main effect of phase. If rewards modulate retention of the adaptation, this would result in an interaction of reward and phase: greater adaptation asymptotes in the retention phases for the spatial & reward group.

The influence of reward on the early adaptation was analyzed in a separate mixed-model ANOVA with reward as a between-subjects factor and repetition as a within-subjects factor. We expected savings, which would result in a main effect of repetition with early adaptation being greater in the second learning phase. Moreover, if rewards result in faster adaptation we would expect a main effect of reward on the early adaptation.

Whether being rewarded or not affected changes in response to spatial errors was analyzed in a mixed-model ANOVA with reward group (spatial only, spatial & reward) as a between-subjects factor and error size (below average, above average) as a within-subjects factor. A separate ANOVA was performed for the trials that were classified as ‘success’ trials and for the trials that were classified as ‘fail’ trials.

## Results

**Fig. 2:**
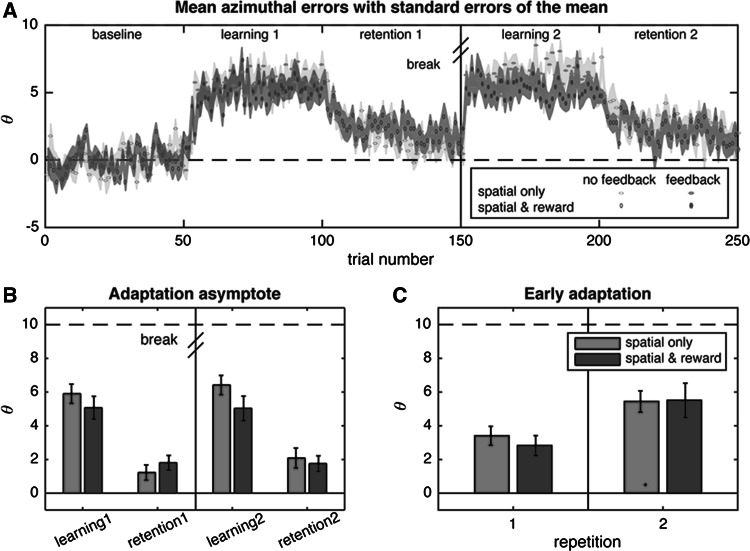
Results. **a** Mean azimuthal error (*θ*) as a function of trial number with *shaded areas* representing standard errors of the mean. *Open symbols* represent trials without feedback, whereas *filled symbols* represent trials with feedback (spatial only or spatial & reward). **b** Mean adaptation asymptotes in the different adaptation phases, with standard errors of the mean. **c** Mean early adaptation in the two learning phases, with standard errors of the mean

On average, 42 % of the trials in the learning phases was rewarded. The reward criterion was relaxed—due to lack of success on 10 consecutive trials—on 2 % of the trials in the learning phases. The mean azimuthal errors as a function of trial number for the two experimental conditions are shown in Fig. 2a.

**Fig. 3:**
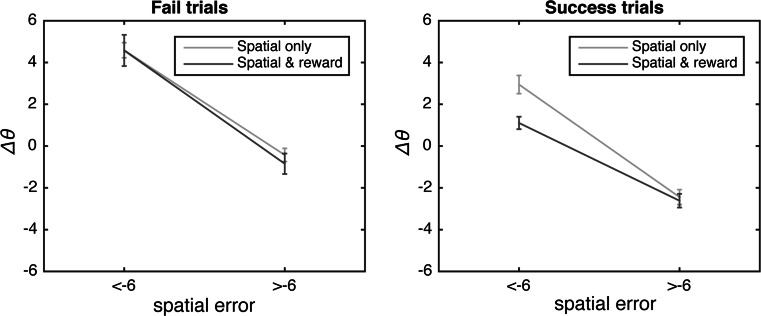
Change in azimuthal error Δ*θ*. *Left panel* Mean Δ*θ* for the ‘fail’ spatial errors smaller or larger than the mean spatial error in the learning phases for the spatial only and spatial & reward group. *Right panel* Mean Δ*θ* for the ‘success’ spatial errors smaller or larger than the mean spatial error in the learning phases for the spatial only and spatial & reward group. *Error bars* represent standard errors of the mean

The mixed-model ANOVA on the adaptation asymptotes showed the predicted main effect of phase: Adaptation asymptotes were higher in learning phases compared to retention phases (*F*(1,38) = 131.21, *p* < 0.001, $\eta_{p}^{2}$ = 0.78). However, there was also no main effect of reward (*F*(1,38) = 0.51, *p* = 0.48, $\eta_{p}^{2}$ = 0.01), and more importantly: there was no interaction of reward and phase (*F*(1,38) = 3.28, *p* = 0.08, $\eta_{p}^{2}$ = 0.08), indicating that the rewards did not influence how much participants adapted their azimuthal errors to the perturbed feedback of how much of the adaptation was retained (Fig. 3).

The mixed-model ANOVA on the early adaptation data also revealed the predicted main effect of repetition (*F*(1,38) = 28.09, *p* < 0.001, $\eta_{p}^{2}$ = 0.43). However, there was no main effect of reward on the early adaptation (*F*(1,38) = 0.77, *p* = 0.78, $\eta_{p}^{2}$ < 0.01), nor did reward and repetition interact (*F*(1,38) = 0.54, *p* = 0.47, $\eta_{p}^{2}$ = 0.01). Thus, rewards did not affect the rate of adaptation.

The ANOVA on Δ*θ* for success trials (Figure 3, left panel) revealed both a main effect of spatial error (*F*(1,38) = 317.48, *p* < 0.001, $\eta_{p}^{2}$ = 0.90) and an interaction of reward group and spatial error (*F*(1,38) = 10.74, *p* = 0.002, $\eta_{p}^{2}$ = 0.23), indicating that Δ*θ* was smaller for success trials that had been rewarded than for success trials that had not been rewarded (spatial only group). The ANOVA on Δ*θ* for the fail trials (Figure 3, right panel), in contrast, revealed a main effect of spatial error (*F*(1,38) = 217.54, *p* < 0.001, $\eta_{p}^{2}$ = 0.85), but no interaction of spatial error and reward group (*F*(1,38) = 0.34, *p* < 0.57, $\eta_{p}^{2}$ = 0.01).

Because movement distance may influence the amplitude of movement errors (Wei and Kording 2009) and the participants’ starting and end positions were unconstrained, we also compared the actual distance that participants moved. A mixed-model ANOVA with reward as a between-subjects factor and phase and repetition as within-subjects factors showed that there was no main effect of or interaction with reward group. Thus, our findings that reward did not influence adaptation were not affected by participants in different reward groups moving different distances between targets. However, participants moved further in the learning phases in which spatial feedback was available (*F* = 20.12, *p* < 0.001, *η* = 0.35; with mean 165.03 mm for the learning phase and 178.71 mm for the retention phase). In addition, subjects moved a bit further in the second repetition compared to the first repetition (*F* = 11.03, *p* < 0.01, *η* = 0.23; with mean 168.73 mm for the first repetition and 175.01 mm for the second repetition).

In an additional exploratory analysis, we checked whether the fact that some participants received financial compensation for participation time whereas other colleagues—that were already paid for being at the university—did not. To test whether the results would have been different if none of the participants were paid, we reran the ANOVAs on the adaptation asymptotes and early adaptation for the selection of participants that received no financial compensation. These ANOVAs revealed no significant interactions of reward group and adaptation phase or repetition.

### Reward only group

**Fig. 4:**
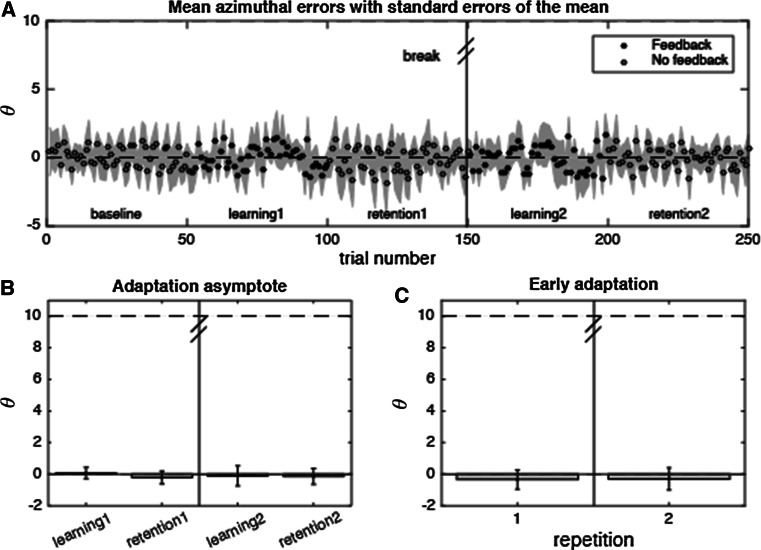
Results of the reward only control experiment (*N* = 6). **a** Mean azimuthal error with shaded standard error of the mean areas as a function of trial number. **b** Mean adaptation asymptote in the different adaptation phases. **c** Mean early adaptation in the two learning phases. *Error bars* represent standard errors of the mean

To analyze whether participants could use the rewards in their adaptation when the rewards were presented without spatial feedback, we analyzed azimuthal errors in the control experiment (Fig. 4a). In this experiment, on average 40 % of the trials in the learning phases was rewarded. In this group, the reward criterion was relaxed—due to lack of success on 10 consecutive trials—on 4 % of the trials in the learning phases. Adaptation asymptotes (Fig. 4b) and early adaptation (Fig. 4c) were compared between adaptation phases using repeated-measures ANOVAs with phase and repetition as within-subjects factors.

A two-way ANOVA on the adaptation asymptotes showed that, in contrast to the findings of experiment 1, there was no main effect of phase [*F*(1,5) = 0.99, *p* = 0.37, $\eta_{p}^{2}$ = 0.17]. Neither was there a main effect of repetition [*F*(1,5) = 0.1, *p* = 0.76, $\eta_{p}^{2}$ = 0.02]. Thus, participants did not adapt their azimuthal errors to the reward feedback when it was presented alone. Consistently, a one-way ANOVA on the early adaptation data showed no effect of repetition [*F*(1,5) = 0.11, *p* = 0.92, $\eta_{p}^{2}$ = 0.002].

## Discussion

In this study, we investigated whether rewarding imperfect performance modulates how participants learn from spatial errors. We investigated this question using a 3D pointing task in which participants adapted to a 10-deg eye-centered rotational perturbation of visual feedback about hand position. Adaptation was compared between two principal groups: a ‘spatial only’ and a ‘spatial & reward’ group. In both groups, a blue feedback cube provided spatial feedback about terminal pointing errors and an adaptive reward criterion was calculated that assigned about half of the trials as ‘success’ trials and half as ‘fail’ trials. Of these groups, only participants in the ‘spatial & reward’ group were rewarded for success trials with a reward sound, target color change and scored points. In addition, we measured a ‘reward only’ group that was rewarded for success trials but saw no spatial errors. Learning and retention of adaptation to the rotational perturbation were assessed in alternating blocks with and without feedback (spatial, reward, or a combination of the two).

In both the spatial only and spatial & reward group, we found the hallmark features of adaptation: learning of corrections to the perturbation, incomplete retention and savings: faster re-learning upon second exposure to the same perturbation. Azimuthal errors increased in response to seeing perturbed spatial feedback and decreased when the feedback was removed without returning to baseline completely (Fernandez-Ruiz et al. 2004; Smith et al. 2006; van der Kooij et al. 2013). In addition, upon second exposure to the perturbed feedback, a higher level of adaptation was reached in the first few trials, which was indicative of faster re-learning, also called savings (Krakauer 2009). In the reward only group, in contrast, there was no adaptation: Azimuthal errors remained stable throughout the learning and retention phases. Thus, participants were unable to use the reward feedback when it was presented alone. A more fine-grained analysis of how the rewards modulated responses to spatial errors on a trial-by-trial basis revealed the most interesting result. We found that the reward feedback modulated adaptive changes to spatial errors: Participants in the spatial & reward group made smaller changes to success trials compared to fail trials, whereas this difference was not present for participants in the spatial only group.

Thus, our main finding is that rewards did not affect the overall outcome of the adaptation, whereas on a trial-by-trial basis, rewards modulated adaptive responses to spatial errors. The finding that rewards modulate adaptive changes is in line with recent findings that rewards modulate the characteristics of subsequent spatial errors: Taylor et al. (2014) found that being rewarded decreases the probability that an aiming strategy is changed, whereas Pekny et al. (2015) found that being rewarded decreases the variability between consecutive movements in a reward-history-dependent manner. The finding that the rewards did not affect the overall outcome of the adaptation, however, contrasts with recent reports that rewards enhance the rate or amount of motor adaptation (Abe et al. 2011; Galea et al. 2015; Nikooyan and Ahmed 2015). In the following paragraphs, we discuss how the absence of an overall effect on the adaptation and trial-by-trial modulation of adaptive changes can be explained by three different proposed linkages between reward-based and (spatial) error-based mechanisms of motor adaptation: modulation of error sensitivity (Nikooyan and Ahmed 2015), dopaminergic enhancement of memory signals (Abe et al. 2011; Galea et al. 2015) and an additive effect of reward-based reinforcement learning (Shmuelof et al. 2012).

First, rewards may modulate error sensitivity (Nikooyan and Ahmed 2015), which is the fraction of an error that is corrected for on the next movement. Error sensitivity was long held to be constant but has recently been found to depend on factors such as error size (Criscimagna-Hemminger et al. 2010; Marko et al. 2012; Wei and Kording 2009) and error history (Herzfeld et al. 2014). The idea that rewards modulate error sensitivity is consistent with our finding that rewards modulated adaptive changes to spatial errors, which were a measure of error sensitivity because they capture the relation between a spatial error and the change in error on the next movement. Reduction in learning rates for successful trials seems contradictory to the finding that the early adaptation—reflecting learning rates—was not reduced in the spatial & reward group. The fact that we did not find reduced early adaptation in the spatial & reward group may have been due to effect of reward on the overall learning rate being small and our analysis of early adaptation lacking power to discriminate fine changes in learning rate.

Error sensitivity is generally discussed in a context of implicit learning processes, but recent studies have emphasized the contribution of explicit processes to adaptation (Benson et al. 2011; Haith et al. 2015; Huberdeau et al. 2015; Keisler and Shadmehr 2010; Mazzoni and Krakauer 2006; Redding and Wallace 2002; Taylor et al. 2014). The rewards may have had an influence on the error sensitivity of such explicit processes rather than on the error sensitivity of implicit processes. In support of an influence of rewards on explicit processes, Taylor and colleagues found that changes in aiming direction were smaller when a movement had been rewarded than when the movement had not been rewarded (Taylor et al. 2014).

Secondly, rewards have been hypothesized to affect adaptation through ‘dopaminergic’ enhancement of memory signals about the adaptation (Abe et al. 2011; Galea et al. 2015), which results in a retention benefit (Galea et al. 2015) or in a consolidation benefit, which becomes apparent after a night sleep (Abe et al. 2011). As we found no retention benefit of providing rewards in addition to spatial errors, our results do not support the idea that there is some general dopaminergic enhancement of memory signals. However, it is possible that albeit our rewards being strong enough to modulate adaptive changes, they were not strong enough to elicit dopaminergic enhancement of memory signals, for instance because our rewards were fictitious rather than financial as in (Abe et al. 2011; Galea et al. 2015) or because they were abundant rather than rare. Alternatively, our spatial errors may not have invoked the learning mechanism that benefits from dopaminergic enhancement. We provided terminal spatial feedback about movement errors, whereas most studies that found retention benefits of providing rewards showed continuous spatial feedback on the adaptation. However, it is unlikely that the absence of a retention benefit was due to providing terminal feedback. In a previous study, we found that although continuous feedback was associated with greater retention than terminal feedback, adaptation to both types of feedback could be described by the same learning mechanism (van der Kooij et al. 2013).

Finally, rewards may have an additive effect on the adaptation in which the effects of implicit error-based and reward-based learning accumulate (Shmuelof et al. 2012). Our results can neither confirm nor reject such a hypothesis. Different forms of implicit reward-based learning have been proposed (Huang et al. 2011; Izawa and Shadmehr 2011; Nikooyan and Ahmed 2015; Therrrien et al. 2015) that all share an important distinction with error-based learning: rather than learning a spatial mapping between sensory and motor information, a relation between actions (movements) and rewards is learned, leading to a bias in movement selection rather than an update of the sensorimotor mapping (Huang et al. 2011; Izawa and Shadmehr 2011; Nikooyan and Ahmed 2015). Our paradigm was unlikely to invoke such ‘reinforcement learning’—as supported by the fact that participants were unable to learn from the reward feedback alone. First, reinforcement learning is held to depend on the repetition of successful movements (Huang et al. 2011), and in our experiment participants moved in a different direction on each trial, whereas in most studies that demonstrated reward-based learning, participants repeatedly moved to a single target (Izawa and Shadmehr 2011; Therrrien et al. 2015). Second, the proportion of rewarded trials was kept constant at on average 40 % by decreasing the reward criterion if participants did well, but also by relaxing the reward criterion if participants had not been successful over a number of trials. This may have caused us to reinforce too many ‘bad’ movements to observe an aiming bias toward the direction imposed by the visual feedback. Therrien et al. (2015), in contrast, did find reward-based learning with a similar reward scheme but used a much simpler task in which participants made repetitive pointing movements to a single target with a supported arm. Moreover, they show that in their study the amount of learning may be explained by a balance between exploration and motor noise, with greater amounts of motor noise dampening learning. Our task in which participants moved with the unsupported arm to a different target on each trial evidently involved a much greater amount of both motor and perceptual noise which may have hampered learning. The finding that the error + reward group did show learning could be explained by the reduction in perceptual noise (by adding vision as an additional information source). Interestingly, all studies that found a consolidation benefit of providing rewards in addition to spatial feedback used reward gradients instead of the binary rewards that we used (Abe et al. 2011; Nikooyan and Ahmed 2015). Moreover, the one other study that measured learning from the reward feedback alone in addition to learning from the combination of reward and spatial feedback found that participants were able to learn from the reward feedback alone (Nikooyan and Ahmed 2015). An interesting possibility is that the reward-based enhancement of retention or consolidation found in other studies have depended on participants being able to learn from the rewards alone and therefore constitutes an additive effect of reward-based learning rather than ‘dopaminergic’ enhancement of memory signals.

## Conclusion

To conclude, rewards modulate adaptive changes to spatial errors. In our paradigm, this did not affect the overall rate or outcome of the adaptation and participants were unable to learn from the rewards when they were presented without spatial feedback. Our findings are not in line with the hypothesis that rewards modulate adaptation through dopaminergic enhancement of memory signals: There was no retention benefit of providing rewards in addition to spatial error feedback. Instead, our results are most consistent with the hypothesis that rewards modulate error sensitivity, a finding that is in line with recent results that error sensitivity is adaptable rather than static and influenced by error size (Criscimagna-Hemminger et al. 2010; Marko et al. 2012) and error history (Herzfeld et al. 2014). Besides modulating error sensitivity, more informative rewards may also have additive effects on adaptation.

## Electronic supplementary material

Below is the link to the electronic supplementary material.

Supplementary material 1 (DOCX 1814 kb)

## Footnotes

1 Note that Δ*θ* is composed of an error correction and a random error drawn from a distribution around a (biased) aimpont. It is therefore possible to find a nonzero Δ*θ* for spatial error of zero when the zero spatial error is caused by a random error drawn from the tail of variability distribution.
