# Supplementary material for: Rewarding imperfect motor performance reduces adaptive changes
Source: Exp Brain Res. 2016 Jan 12;234:1441–50. doi: 10.1007/s00221-015-4540-1 (PMC4851704; doi:10.1007/s00221-015-4540-1)
Supplement: Supplementary file 1 — Supplementary material 1 (DOCX 1814 kb) [file 221_2015_4540_MOESM1_ESM.docx]

**Supplementary figures and analyses**

To check whether the results we found for adaptation to azimuthal errors would be different if we studied absolute errors in which participants had to correct for a combination of an imposed perturbation and natural biases (van der Kooij, et al., 2013), we performed the main analyses for the azimuthal errors also for the absolute errors. If the absolute errors were defined as the distance between the position of the hand-held cube and the target cube, the perturbed spatial feedback would have opposite effects on errors in the azimuthal direction and errors in elevation or distance. Errors in the azimuthal direction would increase due to the azimuthal perturbation whereas errors in elevation and distance would decrease in response to the feedback that was veridical in these dimensions. Therefore we defined the absolute error *U* as the distance between the hand-held cube and the position for which the perturbed feedback would indicate that the hand-held cube and target were aligned. Absolute errors passed a Shapiro-Wilcoxon normality test and were analyzed using analyses of variance.

### S1. Mean absolute errors in the main experiment


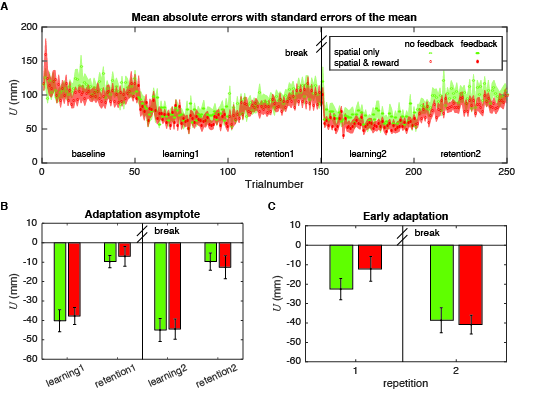


***Figure S.1. A****) Mean absolute error (U) with standard errors of the mean in the different adaptation phases. Data from the spatial only group are plotted in green whereas data from the spatial & reward group are plotted in red.* ***B)****. Mean adaptation asymptote of the absolute errors in the different adaptation phases for the spatial only (green bars) and spatial & reward (red bars) group.* ***C)*** *Mean early adaptation of the absolute error in the learning phases for the spatial only and spatial & reward group.*

A mixed-model ANOVA on the adaptation asymptotes with reward as a between-subjects factor and adaptation phase and repetition as within-subjects factors showed effects similar to those revealed by the ANOVA on the azimuthal errors. There was a main effect of phase (*F*(1,38) = 136.58, *p* < 0.001, *η_p_^2^* = 0.78), indicating that participants adapted their absolute errors to the feedback. However, as we found for the azimuthal errors, there was no interaction of reward and phase *F*(1,38) = 0.08, *p* = 0.78, *η_p_^2^* = 0.01), neither was there a main effect of reward: *F*(1,38) = 0.01, *p* = 0.91, *η_p_^2^* = 0.00). The early adaptation of the absolute errors was analyzed in a mixed-model ANOVA with reward as a between-subjects factor and repetition as a within-subjects factor. As was the case for the azimuthal errors, this analysis revealed savings: a main effect of repetition (*F*(1,38) = 17.79, *p* < 0.001, *η_p_^2^* = 0.32). Again, there was no main effect of reward (*F*(1,38) = 0.42, *p* = 0.52, *η_p_^2^* = 0.1) or interaction of reward and repetition (*F*(1,38) = 1.43, *p* = 0.24, *η_p_^2^* = 0.04).

### S2. Mean absolute errors in the reward only group


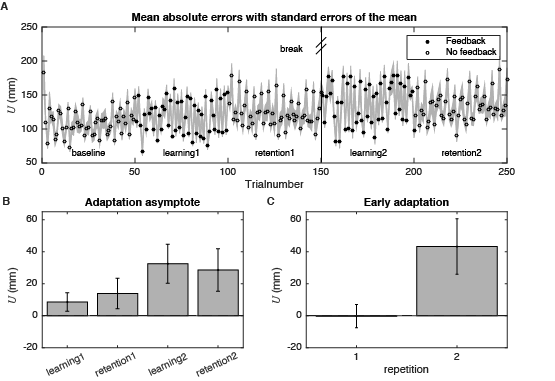


***Figure S2. A****) Mean absolute error (U) in the reward only group with standard errors of the mean in the different adaptation phases.* ***B)****. Mean adaptation asymptote of the absolute error in the different adaptation phases.* ***C)*** *Mean early adaptation in the two learning phases. Error bars represent standard errors of the mean.*

For the reward only group, we also analyzed the mean adaptation asymptote and early adaptation of absolute errors using ANOVAs. As was the case for the azimuthal errors, a repeated measures ANOVA with phase and repetition as within-subjects factors, showed no effect of phase (*F*(1,5) = 0.04, *p* = 0.86, *η_p_^2^* = 0.01) indicating that participants did not adapt their absolute errors to the feedback. However, the ANOVA did show a main effect of repetition (*F*(1,5) = 7.39, *p* = 0.04, *η_p_^2^* = 0.60), indicating that absolute errors increased with repetition, which may have been due to noise in the planning of movements accumulating over time (van Beers, 2009). The fact that we did not see this in the azimuthal errors might have been due to the noise in the movement endpoints being larger in especially the distance component compared to the azimuthal component (van der Kooij, et al., 2013). A one-way ANOVA on the early adaptation of the absolute errors, finally, showed the same main effect of repetition as the ANOVA on the adaptation asymptotes (*F*(1,5) = 7.75, *p* = 0.04, *η_p_^2^* = 0.61) with absolute errors being greater upon repetition of the learning phase.
